# The Protective Effect of *Trichosanthes kirilowii* Peel Polysaccharide on the Oxidative Damaged HepG2 and HUASMC Cells

**DOI:** 10.1155/2022/1792977

**Published:** 2022-07-18

**Authors:** Jinli Zhang, Heren Gao, Liya Zhu, Xiangyu Yuan, Xi Yang, Min Xu, Yang Yang

**Affiliations:** ^1^Anhui Vocational College of Grain Engineering, Hefei, China; ^2^School of Food and Biological Engineering, Hefei University of Technology, Hefei, China; ^3^Key Laboratory of Acupuncture and Moxibustion Foundation and Technology of Anhui Province, Research Institute of Acupuncture and Meridian, College of Acupuncture and Tuina, Anhui Academy of Chinese Medicine, Anhui University of Chinese Medicine, Hefei, China

## Abstract

**Background:**

Oxidative stress is an important cause of liver disease and atherosclerosis. Natural substances with antioxidant activity are good drugs for treating liver disease and atherosclerosis. *Trichosanthes kirilowii* Peel Polysaccharide (TKPP) can remove DPPH (2,2-Diphenyl-1-picrylhydrazyl) free radicals and hydroxyl free radicals *in vitro*, which shows antioxidant activity. Therefore, it is speculated that it can protect human hepatoma cell line (HepG2) and umbilical artery smooth muscle cell (HUASMC) against oxidative damage by hydrogen peroxide (H_2_O_2_).

**Methods:**

Oxidative damage cell models of HepG2 and HUASMC were induced by H_2_O_2_. HepG2 and HUASMC were divided into blank group, H_2_O_2_ injury group, TKPP treatment group, and glutathione (GSH) positive control group. Cell Counting Kit-8 (CCK-8) was used to detect cell viability. The level of total GSH and the amount of Nitric oxide (NO) secreted by cells were detected by specific kits. The gene and protein expressions of catalase (CAT) and superoxide dismutase (SOD) were detected by fluorescence quantitative PCR and Western Blot.

**Results:**

In these two kinds of cells, compared with the control group, the survival rate, total GSH level, and NO secretion, CAT and SOD gene and protein expressions were significantly decreased in the H_2_O_2_ damaged group. In the TKPP treatment group, the cell survival rate was significantly elevated with the increase of the polysaccharide concentration, and the total GSH level, NO secretion, CAT and SOD gene expression, and protein expression levels were also significantly increased.

**Conclusion:**

TKPP can improve the activities of HepG2 and HUASMC cells damaged by H_2_O_2_ and protect the cellular antioxidant system.

## 1. Introduction

According to the previous research, oxidative stress (OS) refers to a condition in which the body's oxidation and antioxidation systems are out of balance, leading to the change and damage of many intracellular molecules like DNA, RNA, lipids, and proteins [1]. OS has a detrimental impact on the body generated by free radicals and reactive oxygen species (ROS) [2], and it is thought to be related to aging [3]. Presently, many studies have shown that OS is involved in the pathogenesis of many chronic diseases [4], including liver diseases and atherosclerosis (AS) [5].

Liver disease and AS have become two seriously harmful diseases that endanger human health. Professor Ceriello introduced the common soil theory in 2004, claiming that OS was the common cause of diabetes, insulin resistance, and heart disease [6], which was proved to be an indisputable fact in 2009 [7]. Based on this theory, substances that have or promote antioxidant functions are considered potential drugs for the prevention and treatment of these diseases. Thus, finding and developing more natural antioxidant substances or their promoting substances are of great significance to the prevention and treatment of liver diseases and AS.

*Trichosanthes kirilowii* Maxim is a kind of perennial climbing herb, born in the mountains and cliffs. According to Chinese traditional medicine, *Trichosanthes kirilowii* Maxim can clear heat and expectoration and nourish the lung to loosen the bowel as an ordinary medicine for a long time [8, 9]. However, its functions in many aspects like cell biology are rarely reported. Recently, it has been reported that the peel of *Trichosanthes kirilowii* Maxim is rich in polysaccharides that can scavenge DPPH free radicals and hydroxyl free radicals in vitro [10]. Therefore, we plan to investigate the role of *Trichosanthes kirilowii* Peel Polysaccharide (TKPP) in antioxidation.

In this paper, TKPP was prepared by the water extraction and alcohol precipitation method. By studying its effects on the cell activity and the secretion of antioxidant substances, such as glutathione (GSH), catalase (CAT), superoxide dismutase (SOD), and nitric oxide (NO) in human umbilical artery smooth muscle cell (HUASMC) and hepatocellular carcinoma (HCC) cell (HepG2) damaged by hydrogen peroxide (H_2_O_2_) oxidation, the antioxidant effect of TKPP was explored. It is hoped to provide a more theoretical basis for the exploration of therapeutic drugs for liver diseases and cardiovascular diseases.

## 2. Materials and Methods

### 2.1. Preparation of TKPP

The fresh *Trichosanthes* skin was dried, shelled, crushed, degreased in order, then added into distilled water at a ratio of 1/20 (m/v), and bathed at 80°C for 2 hours. After the water bath, the material liquid was filtered to remove slag and concentrated, and 4 times anhydrous ethanol was added into the concentrated liquid, and then the volume concentration of ethanol reached 80%. Next, the filtrate was filtered to obtain the residue, which was deproteinized by Sevag method and handled with freeze-drying to obtain TKPP.

### 2.2. Cell Recovery and Passage

HepG2 cells and HUASMC cells were collected from ATCC (Manassas, VA). Human umbilical artery smooth muscle cell HUASMC and hepatocellular carcinoma (HCC) cell HepG2 frozen in a liquid nitrogen tank were taken out and placed in a 37°C water bath to shake and melt. Their cell suspensions were poured into 15 mL centrifuge tubes containing 4 mL DMEM medium (Caisson, North Logan, UT, USA) with 10% FBS (Gendepot, Barker, TX, USA) and centrifuged at 1000 r/min for 3 min to discard the supernatant, then 1 mL 10% FBS and DMEM medium was added, and the suspended cells were blown with suction heads [11]. Next, these cell suspensions were removed to T25 cell culture flask with 4 mL DMEM with 10% FBS and cultured in a carbon dioxide cell incubator with 5% CO_2_ at 37°C. When the cell density in these vials reached about 80%, they were passed on. The old culture medium in each bottle was removed, 5 mL sterile PBS was added for cleaning and drying, and then 1 mL trypsin was added. Cells were placed in the CO_2_ cell culture box for 3 min and observed under the microscope until dispersed suspended cells were digested. Then, 4 mL DMEM with 10% FBS was poured into the culture bottle to terminate trypsin action. The suspension in each bottle was centrifuged at 1000 r/min for 3 min in the centrifuge tube. After discarding the supernatant, 1 mL DMEM with 10% FBS was added, and the suspended cells were gently blown. Next, the required number of cells was added to each cell culture flask, and then DMEM containing 10% FBS was added to make the total volume of the culture medium in each flask become 5 mL. After gently shaking the medium evenly, cell culture was continued in CO_2_ cell culture box with 5% CO_2_ at 37°C.

### 2.3. Cell Viability Detection

Cell viability assay was performed using CCK-8 kit (Dojindo, Shanghai, China). When the density of the above two cells reached about 80%, the culture bottles were taken out, and the suspension cells were achieved by the same operation as the above. The cell concentration was adjusted to 4 × 10^4^ cells/mL, and 100 *μ* L of seeds was absorbed into the 96-well plate for 4 × 10^3^ cells/well. After gentle shaking, the cells were incubated overnight in a CO_2_ cell incubator at 37°C. On the next day, 96-well plates were taken out, and these cells were divided into the blank group (without TKPP and H_2_O_2_), H_2_O_2_ group (negative control group, without TKPP, but with H_2_O_2_), GSH positive control group (with GSH and H_2_O_2_), and TKPP experimental group (with TKPP and H_2_O_2_). In the TKPP experimental group, five concentrations of 0.625 mg/mL, 1.25 mg/mL, 2.5 mg/mL, 5 mg/mL, and 10 mg/mL were set. 2 *μ*·L double steam water was added to the pore plates of both the blank and H_2_O_2_ groups, and 2 *μ*·L 1 mmol/L GSH solution to the pore plates of the GSH positive control group. In TKPP experimental group, 2 *μ*·L of 0.625 mg/mL, 1.25 mg/mL, 2.5 mg/mL, 5 mg/mL, and 10 mg/mL TKPP solutions were added into the cell plate, respectively. The cells of the above treatment groups were placed in the CO_2_ cell culture box and incubated at 37°C for 24 hours. Then, the 96-well plates were taken out, and 2 *μ* L of 300 *μ*·mol/L H_2_O_2_ was added to all groups except the blank group and then incubated in a CO_2_ cell incubator for 4 hours. After that, the supernatant was removed, and 100 *μ*·L DMEM with 10% FBS was added to each well, and 10 *μ*·L CCK-8 solution was supplemented into each well. The cells were placed in a CO_2_ cell culture box for incubation for 2 hours. Finally, a microplate reader (BioTek microplate reader) was applied to measure the optical density (OD) values at 450 nm, and the data were analyzed and processed with Graphpad Prism software.

### 2.4. Total Detection of GSH Level and NO Secretion

HepG2 and HUASMC cells were cultured at 37°C for 24 hours in a 6-well plate with 6 × 10^4^ cells/well. They were still divided into blank group, H_2_O_2_ group (negative control group), GSH positive control group, and TKPP experimental group. The concentration of TKPP in the experimental group was 10 mg/mL. Cells of each group were treated according to the method in 2.3, after that, the medium was removed. Then, cells were washed with PBS twice, and 100 *μ*·L cell lysate was added to each well. The cells and cell lysate were placed on ice for 10 min, collected into a centrifugal tube with a cell scraper, and centrifuged at 12000 r/min for 15 min at 4°C. Also, the supernatant was removed. Finally, the total GSH content of each well was determined according to GSH kit procedure (Sigma), and NO detection kit (Beyotime, Nanjing, China) was used to detect the content of NO in the old cell culture medium removed above. The above data were analyzed and processed by Graphpad Prism software.

### 2.5. RNA Extraction and Quantitative PCR

HepG2 and HUASMC cells were cultured at 37°C for 24 h in a 6-well plate with 6 × 10^4^ cells/well. They were also divided into blank group, H_2_O_2_ group (negative control group), GSH positive control group, and TKPP experimental group, in which 10 mg/mL TKPP concentration was used in TKPP experimental group. Cell treatment in each group was the same as that in 2.3. Then, the culture medium was removed, and cells were washed with PBS twice, 100 *μ*·L Trizol lysis solution (Invitrogen) was added to each well, and cells were placed on ice for 10 min. The total RNA was extracted according to RNA extraction kit, and its OD260/OD280 and concentration were determined by UV spectrophotometer. If the OD260/OD280 size is between 1.8 and 2.0, RNA purity is considered to meet the requirements. Then, the concentration was adjusted to 50 *μ*·g/L, and the mRNA relative expression levels of CAT and SOD were determined by real-time PCR after reverse transcription with 10 *μ*·L reverse transcription reaction system. Reaction conditions were as follows: predenaturation at 95°C for 3 min, denaturation at 95°C for 5 s; annealing at 60°C for 30 s; extending 60°C for 30 s. With GAPDH as an internal reference, PCR primer sequences of each group were shown in Table 1. Relative expression levels of each group were calculated by 2^−△△Ct^ method.

### 2.6. Western Blot (WB) Assay

Cells were plated in 6-well plates with 6 × 10^4^ cells/well and 2 mL culture medium, respectively, and cultured in a cell incubator at 37°C for 24 hours, divided into blank group, H_2_O_2_ group (negative control group), GSH positive control group, and TKPP experimental group. As in 2.3, 300 *μ*·L RIPA cell lysis solution (adding protease inhibitor) was added to each well for ice lysis for 10 min. The lysates were collected and centrifuged at 12000 r/min at 4°C for 10 min, and the supernatant was discarded. Their protein concentrations were determined by BCA method, and then all proteins were quantified to the same concentration. Then, samples were mixed with the loading buffer at a ratio of 1/4, boiled for 10 min, and stored at −20°C. SDS-PAGE electrophoresis was carried out by adding samples with equal amounts of protein to each well. After the electrophoresis, these membranes were transferred by semi-dry method. The required membrane transfer current and time were determined by membrane area and relative molecular weight of protein. After membrane transfer, they were rinsed with PBS for 5 min and then sealed with PBST containing 5% bovine serum albumin at room temperature for 3 hours. Then, they were rinsed 3 times with PBST on a decolorizing shaker for 10 min each, and after that, they were also rinsed with PBST 3 times, 10 min each time. Next, membranes were incubated in secondary antibody diluent at room temperature for 1 hour and rinsed 3 times with PBST for 10 min each time. These membranes were then colored and imaged by ECL chemiluminescence, and the gray level of the bands was analyzed in Image *J*.

### 2.7. Statistical Analysis

At least three parallel samples were used for the experimental data, and the data results were displayed as mean ± standard deviation. Statistical analysis was conducted by Graphpad Prism (GraphPad Prism software, Inc., California, USA). ^*∗*^represents *P* < 0.05, ^*∗∗*^*P* < 0.01, ^*∗∗∗*^*P* < 0.001, and ^*∗∗∗∗*^*P* < 0.0001.

## 3. Results

### 3.1. Different Concentrations of TKPP on Cell Viability

**Figure 1:**
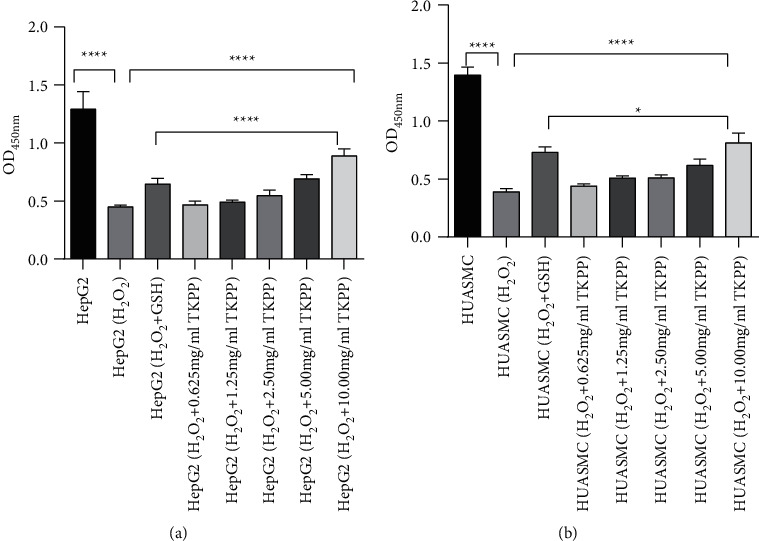
Effects of TKPP with different concentrations on HepG2 (a) and HUASMC (b) cell viability. ^*∗*^*P* < 0.05, ^*∗∗∗∗*^*P* < 0.0001. TKPP could promote the GSH level in H_2_O_2_-damaged cells.

To determine the protective effect of TKPP with different concentrations on oxidative-damaged cells, cell viability was measured with CCK-8. As seen in Figure 1(a) and 1(b), in HepG2 and HUASMC cells, compared with the blank group, the OD value of cells in the H_2_O_2_ group was significantly decreased, suggesting that the oxidative damage cell model was successfully constructed. In GSH group, cell viability was all elevated after adding GSH in the H_2_O_2_ group. Moreover, the addition of TKPP could also elevate cell activity in the H_2_O_2_ group, and the efficiency increased with the increase of TKPP concentration. In this experiment, 10 mg/mL TKPP was the best, and it made cell activity even higher than that of the GSH positive control group. Therefore, 10 mg/mL of TKPP was used in subsequent experimental groups.

**Figure 2:**
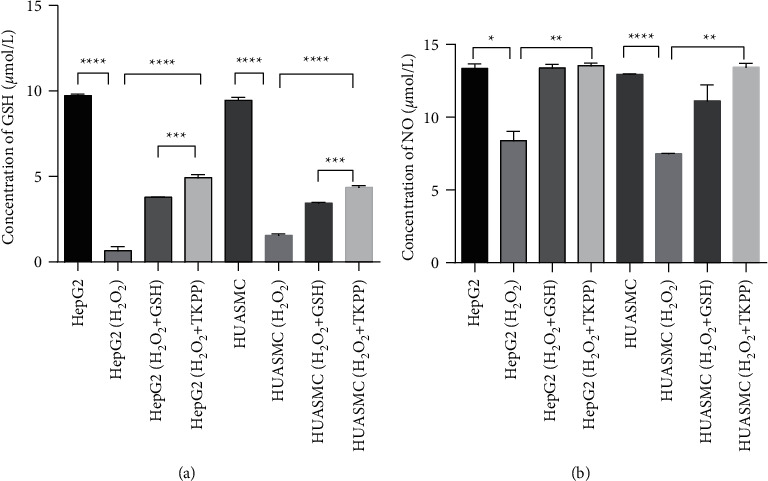
The expression detection of GSH and NO in different groups. (a) The level of GSH in oxidized HepG2 and HUASMC cells. (b) The NO secretion in oxidized HepG2 and HUASMC cells. ^*∗*^*P* < 0.05, ^*∗∗*^*P* < 0.01, ^*∗∗∗*^*P* < 0.001, and ^*∗∗∗∗*^*P* < 0.0001.

In HepG2 and HUASMC cells, the concentration of total GSH decreased significantly in the H_2_O_2_ group compared with the blank group, while the level of total GSH increased significantly in the TKPP group supplemented with 10 mg/mL TKPP. Moreover, the total GSH level of the TKPP group was higher than that of the GSH positive control group (Figure 2(a)). Therefore, we concluded that TKPP effectively increased the levels of total GSH in H_2_O_2_-damaged cells, and it could promote the antioxidant effect in cells.

### 3.2. TKPP Could Effectively Promote the Secretion of NO in H_2_O_2_-Damaged HepG2 and HUASMC Cells

Next, we observed the concentrations of NO in different groups in HepG2 and HUASMC cells to detect the effect of TKPP on NO secretion. It was found that the secretion of NO in the H_2_O_2_ group was significantly decreased compared with the blank group, while the secretion of NO in the TKPP group was significantly increased, and its level was close to that of the GSH positive control group (Figure 2(b)). Thus, we found that TKPP could effectively promote the secretion of NO in H_2_O_2_-damaged HepG2 and HUASMC cells and might resist cell apoptosis, according to NO characteristics [12].

### 3.3. TKPP Could Enhance the Expressions of Antioxidant Enzymes in HepG2 and HUASMC Cells Damaged by H_2_O_2_

**Figure 3:**
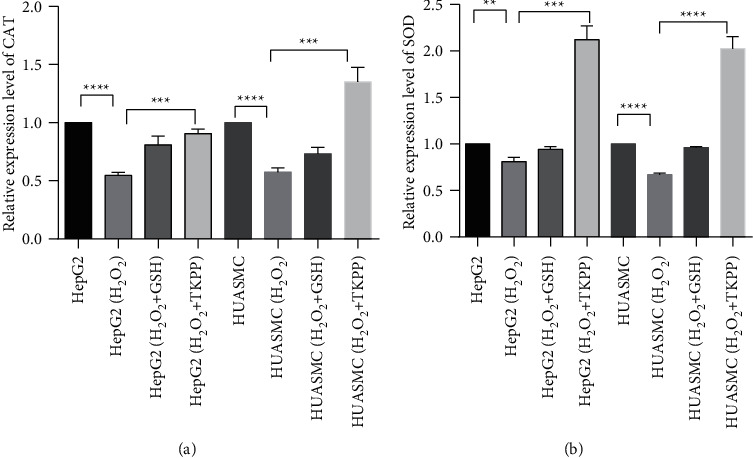
Effects of TKPP on the expression of CAT (a) and SOD (b) in oxidative-damaged HepG2 and HUASMC cells. ^*∗∗*^*P* < 0.01, ^*∗∗∗*^*P* < 0.001, and ^*∗∗∗∗*^*P* < 0.0001. Protein expression detection of CAT and SOD in TKPP group.

To evaluate the effects of TKPP on the antioxidant enzymes in oxidative-damaged HepG2 and HUASMC cells, the levels of CAT and SOD in each group were analyzed by fluorescence quantitative PCR. In both cells, the expressions of CAT and SOD in the H_2_O_2_ group were significantly lower than those in the blank group, while the expression levels of CAT and SOD in the TKPP group were significantly increased when 10 mg/mL TKPP was added. In HepG2 cells, the level of CAT in TKPP group was similar to that in the GSH positive control group, while the level of SOD was much higher. In HUASMC cells, the levels of CAT and SOD genes in TKPP group were also much higher than those in the GSH positive control group (Figure 3(a) and 3(b)). Based on the above findings, TKPP could effectively promote the expressions of antioxidant enzymes in HepG2 and HUASMC cells damaged by H_2_O_2_, thus enhancing the antioxidant effect of cells.

**Figure 4:**
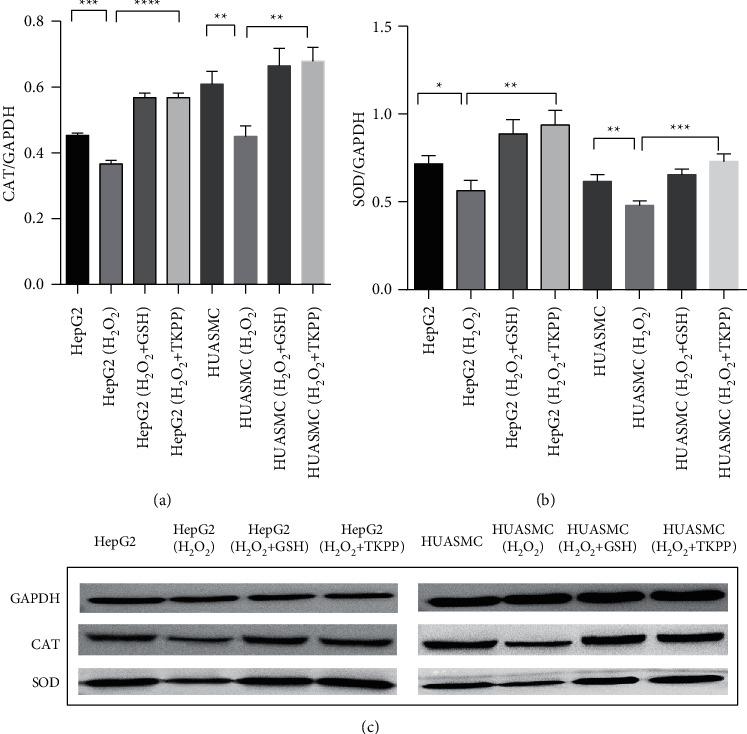
Protein expression detection of CAT and SOD in TKPP group. (a) Band intensity of CAT protein expressions in each group. (b) Band intensity of SOD protein expressions in each group. (c) Immunoblotting of CAT and SOD proteins in each group. ^*∗*^*P* < 0.05, ^*∗∗*^*P* < 0.01, ^*∗∗∗*^*P* < 0.001, and ^*∗∗∗∗*^*P* < 0.0001.

In order to determine the effect of TKPP on the expression of antioxidant enzyme proteins in oxidative-damaged HepG2 and HUASMC cells, the expression of CAT and SOD proteins in each group was analyzed by WB. In both cells, the levels of CAT and SOD proteins in the H_2_O_2_ group were lower than those in the blank group, while in the TKPP group, with the addition of 10 mg/mL TKPP, they were significantly increased, close to or higher than that in the GSH positive control group (Figure 4(a)–4(c)). Thus, polysaccharides from *Trichosanthes kirilowii* Maxim could significantly promote the protein expression of antioxidant enzymes in cells damaged by H_2_O_2_ oxidation.

## 4. Discussion

In biological cells, there exist two antioxidant systems (enzyme and nonenzymatic antioxidant systems) to maintain the balance between oxidation and antioxidation [13]. Enzymatic antioxidants include SOD, CAT, and glutathione peroxidase (GPx); the first two are the most common antioxidant enzymes in living organisms [14]. SOD is the first defense line against oxygen free radicals, which can promote the superoxide into oxygen gas and hydrogen peroxide [15]. When exposed to OS, CAT can be neutralized by the decomposition of hydrogen peroxide into molecular oxygen and water [16]. The expressions of antioxidant enzymes (CAT, SOD, and GPx) can be detected to judge the condition of OS and antioxidant effect in cells.

The nonenzymatic antioxidant system contains GSH, coenzyme *Q*, uric acid, metallothionein, L-carnitine, and so on [17]. These antioxidants can neutralize free radicals and ROS, reduce cell damage caused by them, and prevent oxidative damage to cells and tissues [14]. As a small molecule peptide of three amino acids, GSH is the main reservoir of intracellular sulfo groups and a reducing agent to protect the OS-damaged tissues [18]; thus, it can be used as an indicator of redox potential and the ability of cells to prevent OS. Moreover, it also participates in the detoxification of exogenous compounds and the metabolism of various intracellular compounds in vivo [19]. Herein, we detected the levels of GSH, CAT, and SOD in oxidative-damaged cells to observe the antioxidative efficiency of TKPP. The results demonstrated that TKPP could promote their expressions and possess a good antioxidative ability.

NO is usually catalyzed by nitric oxide synthase (NOS) to produce L-arginine, which exists in various organs like the liver, lung, and blood vessels [20]. It has been reported to have an important role in cell homeostasis, neurotransmission, central nervous system neuroregulation, immune response, signal transduction, cell proliferation, and apoptosis and can effectively combat OS [21]. NO can combine with hydroxyl radical and superoxide anion radical to detoxify as an antioxidant role [22]. In addition, it mediates the expression of cellular protective genes, such as heat shock protein HSP70, to prevent liver cells from the apoptosis induced by TNF*α*, oxygen ions, and nitrite ions [23]. Besides, NO can also indirectly block the activation of the Caspase family by changing mitochondrial permeability, thereby preventing the Caspase activation and blocking the apoptosis signaling, thus inhibiting the cell apoptosis [12]. In this study, we measured the secretion of NO in different groups and found that TKPP could promote NO expression. So, we suspected that TKPP might have an inhibitory function for cell apoptosis, which needed further investigation.

H_2_O_2_ can cause cell peroxidation damage by producing oxygen free radicals or hydroxyl free radicals [24, 25]. Previously, many reports chose the hydrogen peroxide-induced OS cell model to comprehensively evaluate the antioxidant activity of plant chemical components or extracts and elaborate the antioxidant mechanism accordingly [26]. Based on this foundation, we also chose H_2_O_2_ to prepare the oxidation-damaged model. As for the experimental cells, metabolic enzymes in HepG2 cells remain relatively stable, and phenotypes and inner parts do not change with the increase of passage times [27]. It contains biotransforming metabolic enzymes of the same origin as human normal liver parenchyma cells [28] and is often used as an ideal cell line for *in vitro* hepatocyte metabolism and genetic testing [29]. In addition, oxidative damage and abnormal apoptosis of vascular smooth muscle cells can promote the formation of atherosclerotic plaques, thus promoting the development of AS [30, 31]. Therefore, HepG2 and HUASMC cells were selected for oxidative damage experiments to study the antioxidant protection of TKPP.

In our functional experiments, we firstly determined the best concentration of TKPP in the oxidation-damaged cells, and 10 mg/mL showed the best promotion for cell viability. Next, cells were divided into blank group, H_2_O_2_ group, GSH positive control group, and TKPP experimental groups, and the expressions of GSH, NO, SOD, and CAT were detected, respectively. Next, WB was applied to measure the protein expressions of CAT and SOD. All the results showed that TKPP was an antioxidative promotor in oxidation-damaged cells, indicating that it had a good antioxidative effect in cells. In other words, it offers a direction for new drug research and development in many diseases, including liver diseases and AS.

## 5. Conclusion

In summary, TKPP can effectively promote the antioxidant effect of human hepatoma cell line HepG2 and human umbilical artery smooth muscle cells HUASMC and resist oxidative damage and apoptosis induced by H_2_O_2_ by enhancing the expressions of GSH, CAT, and SOD in the natural antioxidant system and the level of NO secretion. Therefore, TKPP has the potential to be the therapeutic target in the prevention and treatment of liver diseases and AS, which is expected to be applied in the clinic in the future.

## Figures and Tables

**Table 1:** Real-time PCR primer sequences.

Gene	Forward (5′–3′)	Reverse (5′-3′)
GAPDH	GGAGCGAGATCCCTCCAAAAT	GGCTGTTGTCATACTTCTCATGG
CAT	TAAGACTGACCAGGGCATC	CAACCTTGGTGAGATCGAA
SOD	GAGATGTTACACGCCCAGATAGC	AATCCCCAGCAGTGGAATAAGG

## Data Availability

The datasets used during the present study are available from the corresponding author upon reasonable request.
